# Supplementary material for: The Evolution and Characterization of the RNA Interference Pathways in Lophotrochozoa
Source: Genome Biol Evol. 2024 May 7;16(5):evae098. doi: 10.1093/gbe/evae098 (PMC11114477; doi:10.1093/gbe/evae098)
Supplement: evae098_Supplementary_Data [file evae098_supplementary_data.zip › Supplementary_materials.zip!/selected_miRNAs/NoGe_selected_miRNAs/mirdeep_pdf_files/NMRB01000076.1_Notospermus_geniculatus_strain_Ushimado_scaffold76,_whole_genome_shotgun_sequence_2792.pdf]

Provisional ID	: NMRB01000076.1_Notospermus_geniculatus_strain_Ushimado_scaffold76,_whole_genome_shotgun_sequence_2792
Score total	: 116.3
Score for star read(s)	: 3.9
Score for read counts	: 111.3
Score for mfe	: 1.7
Score for randfold	: 0
Score for cons. seed	: -0.6
Total read count	: 230
Mature read count	: 123
Loop read count	: 0
Star read count	: 107

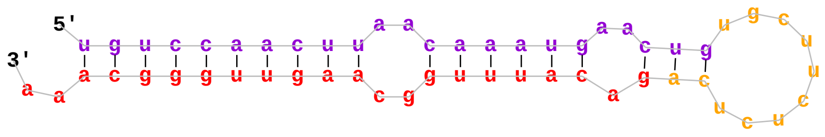

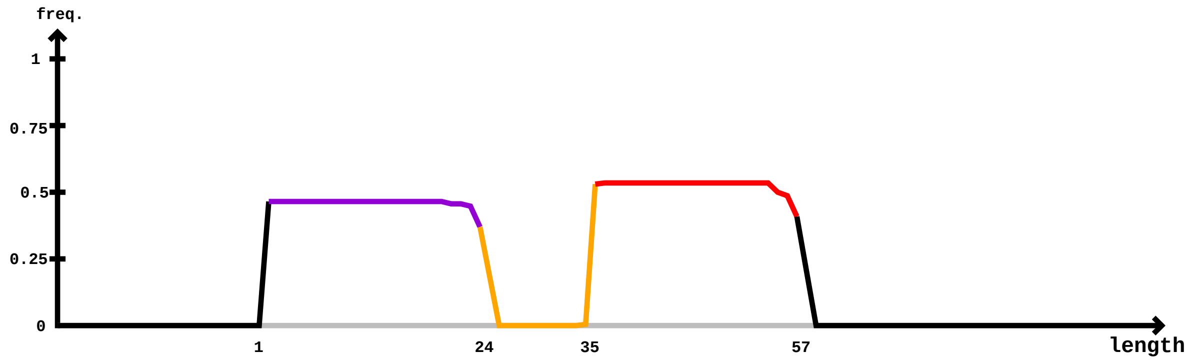

Star **Mature**

```
5'- ggcgucuuggcuggagcaguuguccaacuuaacaaaugaacugugcuucucucagacauuuggcaaguugggcaaaauguuucagacaucaaauauuggcaaccaauauuggu   -3'    obs
    ggcgucuuggcuggagcaguuguccaacuuaacaaaugaacugugcuucucucagacauuuggcaaguugggcaaaauguuucagacaucaaauauuggcaaccaauauuggu          exp
    .......((.((((((((.(((((((((((..((((((..(((.........))).))))))..)))))))))))..)))))))).))...((((((((...))))))))...    reads    mm    sample
    ....................uguccaacuuaacaaauga..........................................................................    1        0     Mm3
    ....................uguccaacuuaacaaaugaac........................................................................    1        0     Mm3
    ....................uguccaacuuaacaaaugaacu.......................................................................    1        0     Mm3
    ....................uguccaacuuaacaaauCaacugu.....................................................................    1        1     Mm3
    ....................uguccaacuuGacaaaugaacugu.....................................................................    1        1     Mm3
    ....................uguccaacuuaacaaaugaacugu.....................................................................    1        0     Mm3
    .....................................................agacauuuggcaaguugggc........................................    1        0     Mm3
    ......................................................gacauuuggcaaguugggcaaa.....................................    1        0     Mm3

    ....................uguccaacuuaacaaaugaacu.......................................................................    7        0     Fm3
    ....................uguccaacuuaacaaaugaacug......................................................................    26       0     Fm3
    ......................................................gacauuuggcaaguugggc........................................    6        0     Fm3
    ......................................................gacauuuggcaaguugggca.......................................    3        0     Fm3
    ......................................................gacauuuggcGaguugggcaa......................................    1        1     Fm3
    ......................................................gacauuuggcaaguugggcaa......................................    16       0     Fm3
    ......................................................gacauuuggcaaguugggcaaG.....................................    1        1     Fm3
    ......................................................gacauuuggcaaguugggcaaa.....................................    50       0     Fm3
    ......................................................gacauuuggcaaguuggCcaaaa....................................    1        1     Fm3
    ......................................................gacauuuggcaaguugggcaaaa....................................    22       0     Fm3
    ......................................................gacauuuggcaaguugggcaaaG....................................    3        1     Fm3
    .......................................................acauuuggcaaguugggcaa......................................    1        0     Fm3

    ....................uguccaacuuaacaaaugaacug......................................................................    18       0     Mm1
    ......................................................gacauuuggcaaguugggcaaa.....................................    6        0     Mm1

    ....................uguccaacuuaacaaauga..........................................................................    1        0     Mm2
    ....................uguccaacuuaacaaaugaac........................................................................    1        0     Mm2
    ....................uguccaacuuaacaaaGgaacu.......................................................................    1        1     Mm2
    ....................uguccaacuuaacaaaugaacu.......................................................................    9        0     Mm2
    ....................uguccaacuuaacaaaugaacug......................................................................    29       0     Mm2
    ....................uguccaacuuaacaaaugaacugu.....................................................................    3        0     Mm2
    ......................................................gacauuuggcaaguugggc........................................    1        0     Mm2
    ......................................................gacauuuggcaaguugggcaaa.....................................    3        0     Mm2
```

```
                    Star                             Mature
ggcgucuuggcuggagcaguuguccaacuuaacaaaugaacugugcuucucucagacauuuggcaaguugggcaaaauguuucagacaucaaauauuggcaaccaauauuggu
......................................................gacauuuggcaaguugggcaaaG....................................    1    1    Mm2
......................................................gacauuuggcaaguugggcaaaa....................................    2    0    Mm2
......................................................gacauuuggcaaguugggcaaaU....................................    1    1    Mm2

....................uguccaacuuaacaaaugaacug......................................................................    4    0    Fm1

....................uguccaacuuaacaaaugaacugu.....................................................................    2    0    Fm2
......................................................gacauuuggcaaguugggcaaa.....................................    3    0    Fm2
```
